# Getting to grips with hepatitis

**DOI:** 10.7554/eLife.00301

**Published:** 2012-11-13

**Authors:** Zhijian J Chen, Jin Ye

**Affiliations:** **Zhijian J Chen** is an *eLife* reviewing editor, and is in the Department of Molecular Biology and the Howard Hughes Medical Institute, University of Texas Southwestern Medical Center, Dallas, United Stateszhijian.chen@utsouthwestern.edu; **Jin Ye** is in the Department of Molecular Genetics, University of Texas Southwestern Medical Center, Dallas, United Statesjin.ye@utsouthwestern.edu

**Keywords:** Sodium taurocholate cotransporting polypeptide, receptor, hepatitis B virus, hepatitis D virus, liver, virus infection, Viruses, Other

## Abstract

The receptor that allows hepatitis B and hepatitis D viruses to enter human liver cells has been identified as a protein that transports bile acids in the liver.

**Related research article** Yan H, Zhong G, Xu G, He W, Jing Z, Gao Z, Huang Y, Qi Y, Peng B, Wang H, Fu L, Song M, Chen P, Gao W, Ren B, Sun Y, Cai T, Feng X, Sui J, Li W. 2012. Sodium taurocholate cotransporting polypeptide is a functional receptor for human hepatitis B and D virus. *eLife* **1**:e00049. doi: 10.7554/eLife.00049

**Image** HepG2 cells infected with the hepatitis B virus

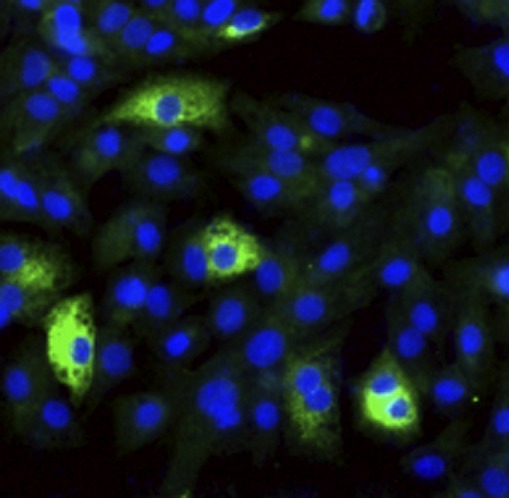

Approximately two billion people around the world are infected with the hepatitis B virus (HBV), and more than 350 million of them are chronic carriers. HBV infection causes hepatitis and liver cirrhosis, and also greatly increases the likelihood of liver cancer (Chisari et al., 2010). Moreover, these liver diseases can be worsened by co-infection with hepatitis D virus (HDV), a satellite virus that can propagate only in the presence of HBV (Makino et al., 1987). Despite the huge success of HBV vaccine, which has dramatically reduced new infections in the developed world, HBV infection is still a major epidemic in developing countries, and current therapies for acute and chronic infections are limited by severe side effects and drug resistance (Kwon and Lok, 2011).

In recent decades there have been significant improvements in our understanding of the viral life cycle, the role of viral proteins in virus replication and assembly, the host immune responses against the virus, and the pathological mechanisms of viral hepatitis and liver cancer. However, there is a major gap in our basic understanding of HBV and HDV—we do not know the identity of the receptor that enables these viruses to enter human liver cells. Now, in *eLife*, Wenhui Li of the National Institute of Biological Sciences in Beijing and co-workers report that they have identified this receptor (Yan et al., 2012).

HBV is a small but remarkable virus that contains a 3.2 kb circular DNA (Figure 1). This DNA serves as a template to produce viral pregenomic and subgenomic RNAs that encode for the following proteins: an envelope protein that comes in three different sizes (large, middle and small), a core protein, a DNA polymerase that also has reverse transcriptase activity, and an X protein that has largely unknown functions. HBV infects humans by binding to a receptor on the surface of hepatocytes (a type of liver cell). HDV is smaller than HBV, and contains RNA rather than DNA, and is believed to enter cells via the same mechanism as HBV.

**Figure 1.**
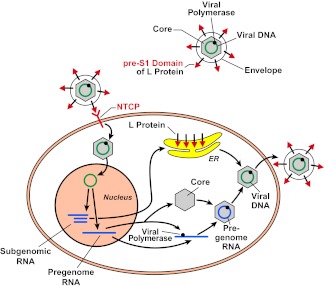
The hepatitis B virus (HBV) contains DNA and polymerase in a viral capsid made by the core protein; this central region is surrounded by a lipid envelope that contains large, middle and small envelope proteins (top right; middle and small proteins not shown). In order to enter a liver cell, the virus binds to a receptor on the surface of the cell through the pre-S1 domain of the large envelope protein (L). Yan, Zhong and co-workers have now shown that a protein known as NTCP is a receptor for HBV. On entering the cell, the DNA inside the virus is transported to the nucleus, where it is converted to covalently closed circular DNA. This DNA serves as a template to produce subgenomic RNA, which encodes for the three envelope proteins: these proteins are synthesized in the endoplasmic reticulum (ER). It also produces pregenomic RNA, which encodes for the core protein and the polymerase, and the polymerase/pregenomic RNA complex is then packaged with the core protein to form the viral capsid (inside which the polymerase converts the pregenomic RNA into viral genomic DNA). This capsid then acquires a lipid envelope containing the viral envelope proteins, and the mature viruses are released from the cells.

After entering the cell via the process of receptor-mediated endocytosis, the HBV is uncoated and the core protein and genomic DNA are transported to the nucleus. This marks the start of a sequence of events that results in the production of viral proteins and the synthesis of viral DNA, which then assemble into mature viral particles that are released from the cells (Figure 1). Although HBV infection itself is not harmful to the infected cells, the expression of HBV proteins in hepatocytes causes the immune system to attack cells that are infected with the virus, which results in hepatitis and other liver pathology (Chisari et al., 2010).

Why has the receptor for HBV and HDV remained elusive for such a long time? A major reason is the technical difficulty in working with these viruses. HBV infects only primary hepatocytes in humans, chimpanzees and a primate-like animal called the treeshrew (*Tupaia belangeri*), but it does not infect other animals such as monkeys, rats, mice or rabbits. So far, no transformed or immortalized cell lines can be infected with HBV. Thus, HBV research is limited by the availability of primary hepatocytes from permissive hosts.

Despite these technical challenges, the Beijing team, which included Huan Yan and Guocai Zhong as joint first authors, was able to identify the HBV/HDV receptor in a tour de force series of experiments. In particular, the researchers maintained a treeshrew animal facility to provide a large supply of primary hepatocytes. They also used deep sequencing to obtain a full record of all the RNA in treeshrew cells, and used this information to build a database of all the proteins found in treeshrews.

It was known that HBV and HDV infection could be blocked by a peptide that contains the same amino acid sequence as a region called the pre-S1 domain in the large envelope protein, and it was thought that this pre-S1 peptide might block infection by binding to the putative viral receptor (Glebe and Urban, 2007). Using a method called zero-distance photo-affinity cross-linking (Suchanek et al., 2005), Yan, Zhong and co-workers were able to isolate the receptor that bound to the pre-S1 peptide, and to identify it as sodium taurocholate cotransporting polypeptide (NTCP, also known as SLC10A1) through mass spectrometry. NCTP is an integral membrane protein normally involved in bile acid transport in the liver (Hagenbuch and Meier, 1994).

Several lines of evidence strongly suggest that NTCP is co-opted by HBV and HDV to enter hepatocytes. Knockdown of NTCP by RNA interference in primary hepatocytes from treeshrews and humans inhibited infection and replication of both viruses. Expression of NTCP in human hepatoma cell lines such as Huh7 and HepG2—which have very low or undetectable expression of NTCP, and are not susceptible to either virus—rendered these cells permissive to infection by both viruses. Furthermore, sequence swapping revealed that replacing a sequence of nine amino acids in NTCP taken from monkeys (which are not susceptible to either virus) with the corresponding sequence from the human form of this protein converts the monkey NTCP into a functional receptor for both viruses.

NTCP is localized to the basolateral plasma membrane of hepatocytes, consistent with its role in capturing blood-borne HBV and HDV. Moreover, the expression of NTCP declined drastically after primary hepatocytes were cultured in vitro, explaining why only freshly isolated hepatocytes were susceptible to HBV infection. Now, armed with the knowledge that NTCP is a receptor for both viruses, researchers can use readily available hepatoma cell lines such as Huh7 and HepG2 to study viral entry, replication and pathogenesis.

The discovery of NTCP as a receptor for HBV and HDV is an important milestone in the fight against hepatitis, but it is by no means the end of the road. Yan, Zhong et al. showed that HBV infection in primary human hepatocytes in vitro yielded very few infectious viral particles. The same was true for hepatoma cell lines that had been engineered to express NTCP. This is in stark contrast to HBV infection in vivo: in both humans and chimpanzees, nearly 100% hepatocytes can be infected and very high viral titers have been reported (Wieland and Chisari, 2005). This means that some factors or conditions that permit highly efficient viral infection and replication in vivo have not been reproduced in the in vitro cell culture system, even in primary hepatocytes. Further work is also needed to confirm that NTCP is a receptor for HBV and HDV in vivo. An ‘acid test’ experiment would be to determine if neutralizing antibodies against NTCP could block HBV and HDV infection in chimpanzees and, eventually, humans.
